# Nanoemulsion as an Effective Treatment against Human-Pathogenic Fungi

**DOI:** 10.1128/mSphere.00729-19

**Published:** 2019-12-18

**Authors:** Alexis Garcia, Yong Yi Fan, Sandeep Vellanki, Eun Young Huh, DiFernando Vanegas, Su He Wang, Soo Chan Lee

**Affiliations:** a South Texas Center for Emerging Infectious Diseases (STCEID), Department of Biology, University of Texas at San Antonio, San Antonio, Texas, USA; b Michigan Nanotechnology Institute for Medicine & Biological Sciences, University of Michigan, Ann Arbor, Michigan, USA; c Department of Internal Medicine, University of Michigan Medical Center, Ann Arbor, Michigan, USA; Hackensack Meridian Health Center for Discovery and Innovation

**Keywords:** *Aspergillus*, *Candida albicans*, *Cryptococcus*, *Mucorales*, fungal infection, nanoemulsion

## Abstract

Infections triggered by pathogenic fungi cause a serious threat to the public health care system. In particular, an increase of antifungal drug-resistant fungi has resulted in difficulty in treatment. A limited variety of antifungal drugs available to treat patients has left us in a situation where we need to develop new therapeutic approaches that are less prone to development of resistance by pathogenic fungi. In this study, we demonstrate the efficacy of the nanoemulsion NB-201, which utilizes the surfactant benzalkonium chloride, against human-pathogenic fungi. We found that NB-201 exhibited *in vitro* activity against Candida albicans, including both planktonic growth and biofilms. Furthermore, treatments with NB-201 significantly reduced the fungal burden at the infection site and presented an enhanced healing process after subcutaneous infections by multidrug-resistant C. albicans in a murine host system. NB-201 also exhibited *in vitro* growth inhibition activity against other fungal pathogens, including *Cryptococcus* spp., Aspergillus fumigatus, and *Mucorales*. Due to the nature of the activity of this nanoemulsion, there is a minimized chance of drug resistance developing, presenting a novel treatment to control fungal wound or skin infections.

**IMPORTANCE** Advances in medicine have resulted in the discovery and implementation of treatments for human disease. While these recent advances have been beneficial, procedures such as solid-organ transplants and cancer treatments have left many patients in an immunocompromised state. Furthermore, the emergence of immunocompromising diseases such as HIV/AIDS or other immunosuppressive medical conditions have opened an opportunity for fungal infections to afflict patients globally. The development of drug resistance in human-pathogenic fungi and the limited array of antifungal drugs has left us in a scenario where we need to develop new therapeutic approaches to treat fungal infections that are less prone to the development of resistance by pathogenic fungi. The significance of our work lies in utilizing a novel nanoemulsion formulation to treat topical fungal infections while minimizing risks of drug resistance development.

## Abstract (precis)

Advances in medicine have resulted in the discovery and implementation of treatments for human disease. While these recent advances have been beneficial, procedures such as solid-organ transplants and cancer treatments have left many patients in an immunocompromised state. Furthermore, the emergence of immunocompromising diseases such as HIV/AIDS or other immunosuppressive medical conditions have opened an opportunity for fungal infections to afflict patients globally. The development of drug resistance in human-pathogenic fungi and the limited array of antifungal drugs has left us in a scenario where we need to develop new therapeutic approaches to treat fungal infections that are less prone to the development of resistance by pathogenic fungi. The significance of our work lies in utilizing a novel nanoemulsion formulation to treat topical fungal infections while minimizing risks of drug resistance development.

## INTRODUCTION

During recent decades, there has been an exponential growth in discoveries and medical advances for the treatment of human disease. This has led to better treatment for patients, and as a result we have been able to prolong human life. While these recent medical advances have certainly been beneficial overall, procedures such as solid-organ transplants and cancer treatments have left many patients in an immunocompromised state. The emergence of immunocompromising diseases, such as HIV/AIDS or other immunosuppressive medical conditions, has opened an opportunity for fungal infections to plague patients globally (1–4).

Candida albicans is a human commensal fungus found on the skin, in mucosal membranes, and in the normal gut flora (5, 6). C. albicans is known to be an opportunistic fungus and the most common fungal pathogen that typically infects immunocompromised patients (3, 4). Treatment for candidiasis currently relies on three major classes of antifungal drugs, including echinocandins, azoles, and polyenes (7, 8). Prior to the introduction of echinocandins, fluconazole was the most common drug used to treat C. albicans infections (7). Recently there has been an increase in cases of drug-resistant C. albicans infections, resulting in an increase in the morbidity and mortality of patients (4, 9–11). One explanation for drug resistance is the development of mutations in the target genes of the antifungal drug (9). Second, the overexpression of efflux pumps and multidrug resistance genes could also lead to antifungal drug resistance (9). In addition, pathogenic fungi can also form biofilms that are resistant to antifungal drugs (12, 13). Thus, it is of upmost importance to develop new therapeutic approaches that are less prone to the development of resistance by pathogenic fungi.

Nanoemulsions (NE) that mechanically disrupt microbial membranes have been developed to control pathogenic bacteria (14, 15). One example is the nanoemulsion NB-201, which is an emulsification of refined soybean oil, water, glycerol, EDTA, Tween 20, and the surfactant benzalkonium chloride (BZK), which is commonly used as an antimicrobial preservative in drugs, topical antiseptic, clinical disinfectant, and sanitation agent in the food industry (14, 16–18). The NB-201 formulation exhibited *in vitro* growth inhibition activity against Pseudomonas aeruginosa and *in vivo* activity in a burn wound animal model. Treatment of animals infected with Staphylococcus aureus with NB-201 by using a murine skin abrasion wound model demonstrated the therapeutic potential of this nanoemulsion formulation (19, 20). NB-201 has also shown activity against multidrug-resistant S. aureus (MRSA) in a study using a porcine burn wound model (14). The NB-201 formulation is also able to greatly reduce the inflammation of infected wounds and is nontoxic to the skin, making it a viable method of treatment for topical bacterial infections (14). More importantly, NB-201 was found to be nontoxic in a porcine wound infection model even after continuous treatment for 20 days (14). In this study, we present the utilization of NB-201 to control pathogenic fungi. NB-201 exhibited *in vitro* efficacy against C. albicans planktonic cells and biofilms.

## RESULTS

### *In vitro* activity against Candida albicans planktonic cells and preformed biofilms.

C. albicans isolates, including antifungal drug-resistant strains (Table 1), were tested. Ninety-six-well plates were inoculated with each C. albicans strain and the NB-201 nanoemulsion (NE) (10%), added in ratios ranging from 1:1 to 1:2,048, as diluted in phosphate-buffered saline (PBS). Minimum fungicidal concentrations (MFCs) were determined by using a 100% killing point of the C. albicans planktonic cells collected at 1, 24, 48, and 72 h after addition of NB-210 to the media (Table 2). We observed, within 1 h, a concentration of 1:512 of the NE was able to kill all the planktonic cells plated. As the incubation time was increased, we observed a lower MFC was required. At 24 h, a concentration of 1:1,024 was able to kill all strains plated. Within 48 h, the concentration of NB-201 required to kill all ten strains remained at an MFC of 1:1,024. At 72 h, the MFC for 100% killing of the strains plated was lowered to a concentration of 1:2,048 (Table 2).

**TABLE 1:** Fungal pathogens used in this study

Species	Strain	Information	Isolation
*Candida albicans*	SC5314	Wild type	Patient with generalized candidiasis
*Candida albicans*	TW1	Clinical isolate (no mutation)	Patient with oropharyngeal candidiasis
*Candida albicans*	TW2	Drug resistance observed (*MDR1*)	Patient with oropharyngeal candidiasis
*Candida albicans*	TW3	Drug resistance observed (*MDR1*)	Patient with oropharyngeal candidiasis
*Candida albicans*	TW17	Multidrug resistance observed (*CDR1*, *MDR1*, *ERG11*)	Patient with oropharyngeal candidiasis
*Candida albicans*	4639	F449S, T229A (Erg11p substitutions), *MDR1*, *CDR1*	Patient with oropharyngeal candidiasis
*Candida albicans*	6482	D116E, K128T, Y132H, D278N, G464S, P230L (Erg11p substitutions)	Patient with oropharyngeal candidiasis
*Candida albicans*	4617	F449S, T229A (Erg11p substitutions)	Patient with oropharyngeal candidiasis
*Candida albicans*	3731	F126L, K143R (Erg11p substitutions), *MDR1*	Patient with oropharyngeal candidiasis
*Candida albicans*	2240	V437I (Erg11p substitutions), *MDR1*, *ERG11*	Patient with oropharyngeal candidiasis
*Candida albicans*	412	K128T (Erg11p substitution)	Patient with oropharyngeal candidiasis
*Cryptococcus neoformans*	H99	Serotype A	Cerebrospinal fluid (CSF) culture of a patient infected with cryptococcosis
*Cryptococcus neoformans*	R265	Serotype B	Patients with cryptococcosis during the Vancouver outbreak cases
*Cryptococcus neoformans*	WSA87	Serotype C	CSF culture from a patient, the NIH culture collection of June Kwon-Chung
*Cryptococcus neoformans*	R4247	Serotype D	CSF culture from a patient, culture collection of the University of Texas Health Science Center in San Antonio Fungus Testing Laboratory (UTHSCSA FTL)
*Aspergillus fumigatus*	CEA10	Wild type, MAT1-1, clinical isolate	Patient with invasive aspergillosis
*Aspergillus fumigatus*	SRRC2006	Laboratory reference strain	Reference strain for identification and morphological observation (ICPA)
*Aspergillus fumigatus*	V044-58	Azole-resistant clinical isolate	Patient with chronic pulmonary aspergillosis
*Aspergillus fumigatus*	F14946	Azole-resistant clinical isolate	Patient with chronic pulmonary aspergillosis
*Aspergillus fumigatus*	F13747	Azole-resistant clinical isolate	Patient with chronic pulmonary aspergillosis
*Aspergillus fumigatus*	F14532	Azole-resistant clinical isolate	Patient with chronic pulmonary aspergillosis
*Aspergillus fumigatus*	F13746	Azole-resistant clinical isolate	Patient with chronic pulmonary aspergillosis
*Aspergillus fumigatus*	F12776	Azole-resistant clinical isolate	Patient with chronic pulmonary aspergillosis
*Aspergillus fumigatus*	F14403	Azole-resistant clinical isolate	Patient with chronic pulmonary aspergillosis
*Aspergillus fumigatus*	F6919	Azole-resistant clinical isolate	Patient with chronic pulmonary aspergillosis
*Aspergillus fumigatus*	F16216	Azole-resistant clinical isolate	Patient with chronic pulmonary aspergillosis
*Rhizopus delemar*	DI18-58	Clinical isolate	Lower lobe of the right lung from a patient with mucormycosis, UTHSCSA FTL
*Rhizopus delemar*	DI18-59	Clinical isolate	Abscess drainage of the right arm from a patient with mucormycosis, UTHSCSA FTL
*Rhizopus microsporus*	DI18-62	Clinical isolate	Wound on the left stump from a patient with mucormycosis, UTHSCSA FTL
*Rhizopus microsporus*	DI18-63	Clinical isolate	Right lung from a patient with mucormycosis, UTHSCSA FTL
*Mucor circinelloides*	DI18-65	Clinical isolate	Wound from a patient with mucormycosis
*Mucor circinelloides*	DI18-66	Clinical isolate	Wound on the right hand of a patient with mucormycosis, UTHSCSA FTL
*Cunninghamella*	DI18-68	Clinical isolate	Pleural tissue of a patient with mucormycosis, UTHSCSA FTL
*Cunninghamella*	DI18-69	Clinical isolate	Wound on the leg of a patient with mucormycosis, UTHSCSA FTL
*Lichtheimia*	DI18-71	Clinical isolate	Pectoralis muscle of a patient with mucormycosis, UTHSCSA FTL
*Lichtheimia*	DI18-72	Clinical isolate	Hard palate of a patient with mucormycosis, UTHSCSA FTL

**TABLE 2:** MFCs of NB-201 on pathogenic fungi

Species (no. of strains)	MIC after:			
	1 h	24 h	48 h	72 h
*Candida albicans* (10)	1:512	1:1,024	1:1,024	1:2,048
*Aspergillus fumigatus* (10)	1:16	1:128	1:512	1:512
*Cryptococcus neoformans* (4)	1:1,024	1:2,048	1:2,048	1:2,048
*Rhizopus delemar* (2)	1:64	1:256	1:512	1:512
*Rhizopus microsporus* (2)	1:4	1:1,024	1:1,024	1:1,024
*Mucor circinelloides* (2)	1:32	1:64	1:512	1:512
*Cunninghamella* (2)	1:32	1:256	1:256	1:256
*Lichtheimia* (2)	1:4	1:256	1:512	1:512

**FIG 1:**
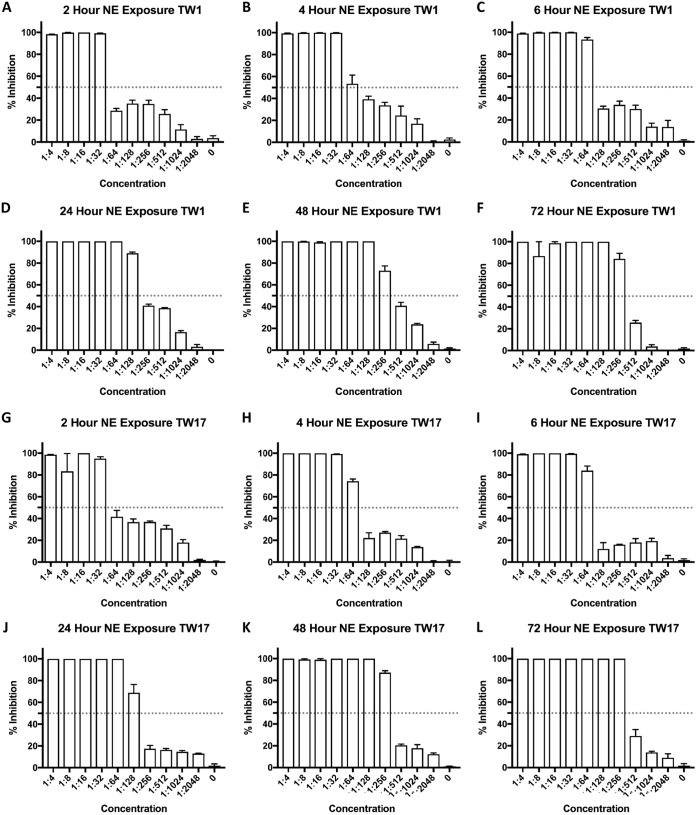
Measurement of metabolism in C. albicans drug-resistant TW1 clinical isolate preformed biofilms. Multidrug-resistant C. albicans clinical isolates TW1 and TW17 were plated in a 96-well plate containing RPMI medium at a concentration of 1 × 10^6^ and incubated for 24 h to form a biofilm on the bottom of the wells. The medium containing the nanoemulsion treatment was then removed, followed by the biofilms being treated with XTT solution, and quantified. (A) Two hours posttreatment with NB-201; (B) 4 h posttreatment with NB-201; (C) 6 h posttreatment with NB-201; (D) 24 h posttreatment with NB-201; (E) 48 h posttreatment with NB-201; (F) 72 h posttreatment with NB-201; (G) 2 h posttreatment with NB-201; (H) 4 h posttreatment with NB-201; (I) 6 h posttreatment with NB-201; (J) 24 h posttreatment with NB-201; (K) 48 h posttreatment with NB-201; (L) 72 h posttreatment with NB-201.

The ability of C. albicans to form biofilms, which increases antifungal drug resistance, is a major virulence factor observed in the clinical setting (12, 13). To test the efficacy of NB-201 on C. albicans biofilms, two multidrug-resistant clinical isolates, TW1 and TW17 (21), were chosen. The C. albicans clinical isolates TW1 and TW17 were plated on 96-well plates and allowed to form a biofilm over the course of 24 h (PFB). We then treated these PFBs with the NE added in various ratios, ranging from 1:1 to 1:2,048, followed by a second-generation tetrazolium (XTT) metabolic assay (Sigma-Aldrich) (22) to measure the ratio of the metabolism, indicative of disruptions of the biofilms, after NB-201 treatments. Within 2 h, an NE concentration of 1:32 was able to inhibit 100% of the metabolism in the TW1 clinical isolate (Fig. 1A). At 4 h, a concentration of 1:64 was inhibiting greater than 50% of the metabolism in the PFBs (Fig. 1B), while incubation with NB-201 at 6 h exhibited higher inhibition, up to 90%, at the same concentration (Fig. 1C). At 24 h, a concentration of 1:128 presented 90% inhibition of metabolism in the TW1 PFBs (Fig. 1D), while a concentration of 1:256 presented 75% metabolism inhibition at 48 h (Fig. 1E). After 72 h of exposure to NB-201, a concentration of 1:256 presented 85% inhibition of the metabolism of the PFBs (Fig. 1F). A similar trend was observed with the TW17 isolate (Fig. 1G to L). A similar result also was obtained to measure the efficacy of NB-201 in the disruption of C. albicans biofilms by using crystal violet staining in the TW1 (see Fig. S1 in the supplemental material) and the TW17 (Fig. S2) isolates. Two independent experiments were performed with similar results, with each experiment containing biological repeats.

10.1128/mSphere.00729-19.1 FIG S1 Crystal violet staining of PFBs from the C. albicans azole-resistant clinical isolate TW1. C. albicans clinical isolate TW1 was plated in 96-well plates and allowed to form a biofilm over the course of 24 h. A crystal violet stain was added, and biofilm mass was then quantified. (A) Two hours after NB-201 addition; (B) 4 h after NB-201 addition; (C) 6 h after NB-201 addition; (D) 24 h after NB-201 addition; (E) 48 h after NB-201 addition; (F) 72 h after NB-201 addition. As incubation time with NB-201 increased, an increase in the disruption of biofilms could be observed. Download FIG S1, JPG file, 0.6 MB. Copyright © 2019 Garcia et al. 2019 Garcia et al. This content is distributed under the terms of the Creative Commons Attribution 4.0 International license.

10.1128/mSphere.00729-19.2 FIG S2 Crystal violet staining of PFBs from the C. albicans azole-resistant clinical isolate TW17. C. albicans clinical isolate TW17 was plated in 96-well plates and allowed to form a biofilm over the course of 24 h. A crystal violet stain was added, and biofilm mass then was quantified. (A) Two hours after NB-201 addition; (B) 4 h after NB-201 addition; (C) 6 h after NB-201 addition; (D) 24 h after NB-201 addition; (E) 48 h after NB-201 addition; (F) 72 h after NB-201 addition. In accordance with our previous TW1 experiment, we observed that as incubation time with NB-201 increased, an increase in the disruption of biofilms could be observed. Download FIG S2, JPG file, 0.6 MB. Copyright © 2019 Garcia et al. 2019 Garcia et al. This content is distributed under the terms of the Creative Commons Attribution 4.0 International license.

### *In vivo* activity of NB-201 against Candida albicans subcutaneous infection.

**FIG 2:**
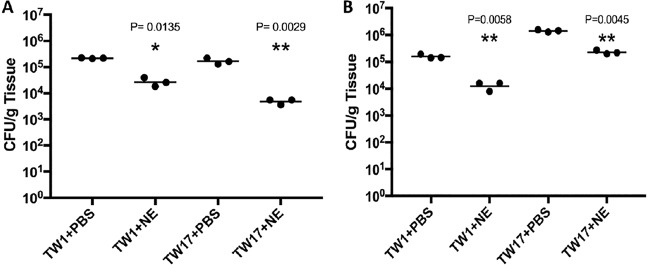
*In vivo* efficacy of NB-201 via subcutaneous infection. *In vivo* efficacy of NB-201 was tested by using a murine subcutaneous infection model. Mice were infected subcutaneously with the multidrug-resistant C. albicans isolate TW1 or TW17. We then treated the mice with NB-201 via subcutaneous injection. To measure fungal burden in the tissues, we plated the homogenized tissue onto YPD agar plates and counted the CFU. (A) Initial infection. A significant reduction in fungal burden can be observed in mice treated with NE in both TW1 (*P* = 0.0135) and TW17 (*P* = 0.0029). (B) Recovered strain infection. Mice were infected with strains recovered from the initial infection to check for development of resistance to the NE. A significant reduction in the fungal burden can be observed in mice treated with NB-201 in both TW1 (*P* = 0.0058) and TW17 (*P* = 0.0045).

**FIG 3:**
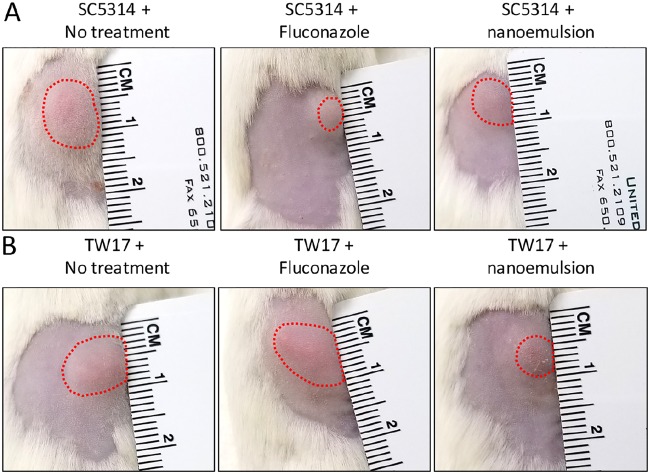
Comparison of NB-201 efficacy to fluconazole via subcutaneous infection with wild-type C. albicans. Comparison of the *in vivo* efficacy of NB-201 to that of fluconazole was tested by using a murine subcutaneous infection model. Mice were infected subcutaneously with the wild-type C. albicans (SC5314) or with the fluconazole-resistant C. albicans (TW17). Subsequent treatments via injection of either fluconazole or NB-201 were monitored for 72 h. (A) Due to SC5314 being susceptible to fluconazole, both fluconazole and NB-201 presented a reduction of swelling (red dashed lines) and inflammation compared to the untreated control. (B) TW17 is intrinsically resistant to fluconazole; thus, no reduction was observed in mice treated with the azole. NB-201 presented a greater reduction of swelling and inflammation. Images are representative of 72 h postinfection.

Nanoemulsions lyse if administered intravenously. Thus, *in vivo* efficacy of NB-201 was tested by using a murine subcutaneous infection model. This model is appropriate to show the NE can spread through the tissue and control pathogenic fungi. Mice were infected subcutaneously with the multidrug-resistant C. albicans isolate TW1 or TW17. We then treated the mice with NB-201 via subcutaneous injection (see Materials and Methods). After 2 days of treatment with NB-201, we euthanized the mice and collected tissues at the site of infection. To measure fungal burden in the tissues, we plated the homogenized tissue onto YPD agar plates and counted the CFU. Treatment with NB-201 resulted in a significant decrease of fungal CFU in mice infected with TW1 (*P* = 0.013) and with TW17 (*P* = 0.002) compared to that of PBS-treated control groups (Fig. 2A). The C. albicans strains from the first infection experiment were recovered and used for a second round of the assay. In the group of mice infected with the recovered TW1 or TW17, we observed a significant decrease of CFU in the tissues of mice treated with the NE (*P* = 0.005 or *P* = 0.004, respectively) compared to mice treated with PBS (Fig. 2B). These results demonstrate that NB-201 has the efficacy to control C. albicans infections, while resistance and/or tolerance against NB-201 is less likely to develop. The azole fluconazole still remains one of the first drugs administered for the treatment of candidiasis (23). With this in mind, we wanted to compare the efficacy of NB-201 to that of fluconazole. In a fashion similar to that of our previous experiment, we began by using a murine subcutaneous infection model. Mice were infected with either the wild-type C. albicans SC5314 or the fluconazole-resistant strain TW17, followed by treatment with either a mock injection (PBS), NB-201, or fluconazole over the course of 72 h. Treatment of NB-201 shows a reduction in swelling and inflammation at the infection site compared to treatment with fluconazole despite the WT strain being susceptible to fluconazole (Fig. S4A). A similar result with the NB-201 treatment can be observed in the mice infected with the azole-resistant strain TW17, with the exception of fluconazole showing no signs of treatment (Fig. 3).

**FIG 4:**
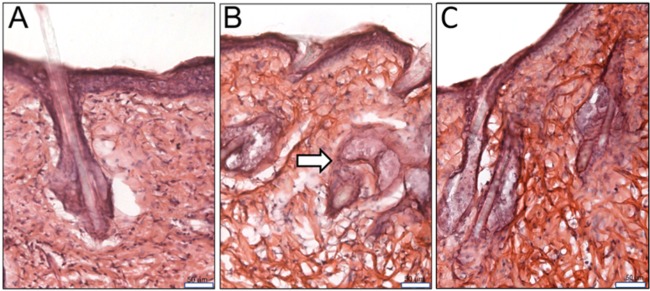
Histopathological analysis of mouse skin tissue postsubcutaneous infection and treatment with NB-201. Samples from uninfected mice, mice infected with C. albicans, and mice infected and treated with NB-201 were sectioned. We then stained our tissue samples with a hematoxylin and eosin stain. (A) Uninfected mouse skin tissue. (B) Infected and untreated mouse skin tissue. Accumulation of infiltrates at the hair follicles can be observed (white arrow). (C) After treatment with NB-201, we observed a reduction in the infiltration of cells within the deep dermis, superficial fat, and hair follicles. Scales are 50 μm in panels A and B.

Following our subcutaneous model, we took tissue samples from uninfected mice, mice infected with C. albicans TW17, and mice infected and treated with NB-201. We then stained our tissue samples with a hematoxylin and eosin stain. Compared to the noninfected mice (Fig. 4A), C. albicans postinfection tissue presents a large collection of infiltration around the hair follicles, deep dermis, and superficial fat (Fig. 4B). After treatment with NB-201, we observed a reduction in the infiltration of cells within the deep dermis, superficial fat, and hair follicles (Fig. 4C). In the untreated tissue, the layers of the skin are not as clearly defined as those of the uninfected tissue. Treatment with NB-201 resulted in the layers of the skin being more defined than the untreated tissues. These observations further support the efficacy of the nanoemulsion to control multidrug-resistant C. albicans infections.

### *In vitro* activity of NB-201 against other pathogenic fungi.

The formulation of NB-201 was further tested to examine its ability to kill other pathogenic fungi (Table 1) in a fashion similar to what was observed in C. albicans. We approached this by inoculating 96-well plates with various fungal strains and added NB-201 in ratios ranging from 1:1 to 1:2,048. We then measured the minimum fungicidal concentrations (MFC) by using a 100% killing point of the fungal strains collected at 1, 24, 48, and 72 h after addition of NB-210 to the medium (Table 2).

### Aspergillus fumigatus.

We performed a checkerboard assay with ten different strains of Aspergillus fumigatus, including drug-resistant strains, all of which are known clinical isolates (Table 1). Within 1 h, we observed that a concentration of 1:16 showed complete killing of all clinical isolates. We note that a concentration of 1:128 was able to kill seven out of the ten A. fumigatus clinical isolates within the same time period (Table 2). As incubation time with the NE progressed, we observed a reduction in the MFC required to kill all of the A. fumigatus clinical isolates. Within 24 h, a concentration of 1:128 showed 100% killing of these clinical isolates (Table 2). Finally, at 48 and 72 h, all ten of the A. fumigatus clinical isolates were killed at a concentration of 1:512 (Table 2).

### Mucorales.

We tested ten clinical isolates of various *Mucorales* species (Table 1). One hour after incubation with NB-201, we observed a total MFC of 1:64 in *Rhizopus delemar* isolates, 1:4 in R. microsporus isolates, 1:32 in Mucor circinelloides isolates, 1:32 in *Cunninghamella* isolates, and 1:4 in *Lichtheimia* isolates (Table 2). In a fashion similar to what we have observed thus far, longer NE incubation with the fungi resulted in a lowered MFC. At 24 h, *R. delemar*, *Cunninghamella*, and *Lichthemia* isolates presented an MFC of 1:256. R. microsporus isolates had an MFC of 1:1,024, while *M. circinelloides* showed an MFC of 1:64 (Table 2). At 48 h *R. delemar*, *M. circinelloides*, and *Lichthemia* isolates resulted in a lowered MFC of 1:512. *Cunninghamella* isolate MFC remained at 1:256, followed by R. microsporus, presenting an unchanged MFC of 1:1,024. At 72 h, we observed no changes in the MFC with any of the *Mucorales* strains (Table 2).

### Cryptococcus neoformans.

Four different serotypes of C. neoformans were tested against NB-201 (Table 1). Within 1 h, an MFC of 1:1,024 was able to kill all four serotypes of C. neoformans (Table 2). This was followed by 24, 48, and 72 h showing an MFC of 1:2,048 (Table 2). NE-treated and untreated C. neoformans samples were plated in medium containing propidium iodide, which is only able to penetrate the cellular membranes of dead or dying cells. Within 30 min of incubation, a clear distinction between live and dead cells can be observed (Fig. S3A), with the dead cells fluorescing red due to the propidium iodide stain (Fig. S3B).

10.1128/mSphere.00729-19.3 FIG S3 Cryptococcus neoformans cell death by NB-201. NE-treated and untreated C. neoformans samples were plated in medium containing propidium iodide, which is only able to penetrate the cellular membranes of dead or dying cells. Within 30 min of incubation, a clear distinction between live and dead cells can be observed. (A) Top, differential interference contrast (DIC) image of untreated C. neoformans cells. Bottom, DIC image of C. neoformans cells treated with NB-201. (B) Top, fluorescent image of untreated C. neoformans cells. Bottom, fluorescent image of C. neoformans cells treated with NB-201. Dead cells fluoresce red due to the propidium iodide stain. Download FIG S3, JPG file, 0.2 MB. Copyright © 2019 Garcia et al. 2019 Garcia et al. This content is distributed under the terms of the Creative Commons Attribution 4.0 International license.

10.1128/mSphere.00729-19.4 FIG S4 Comparison of efficacy of NB-201 and fluconazole via subcutaneous infection with wild-type C. albicans. Comparison of the *in vivo* efficacy of NB-201 to that of fluconazole was tested by using a murine subcutaneous infection model. Mice were infected subcutaneously with the wild-type C. albicans (SC5314) or with the fluconazole-resistant C. albicans (TW17). Subsequent treatments via injection of either fluconazole or NB-201 were monitored for 72 h. Due to SC5314 being susceptible to fluconazole, a reduction was observed in mice treated with the azole. Compared to the azole-treated mice, NB-201 presented a greater healing ability by showing a reduction of swelling and inflammation over the course of 72 h. TW17 is intrinsically resistant to fluconazole; thus, no reduction was observed in mice treated with the azole. Compared to azole treatment of mice, NB-201 treatment presented a greater reduction of swelling and inflammation over the course of 72 h. Download FIG S4, JPG file, 0.5 MB. Copyright © 2019 Garcia et al. 2019 Garcia et al. This content is distributed under the terms of the Creative Commons Attribution 4.0 International license.

## DISCUSSION

Previously, NB-201 presented a highly effective antimicrobial potential against various methicillin-resistant S. aureus strains, both *in vitro* and *in vivo*, by using a porcine wound infection model (14). NB-201 is composed of high-energy droplets that fuse nonspecifically with lipids in the microbial outer membranes, resulting in membrane damage and killing of the organism (14). The surfactant in NB-201, benzalkonium chloride (BZK), is a known biocide found in many over-the-counter antibacterial handwipes, antiseptic creams, and other medically relevant consumer products (24). The use of this surfactant in NB-201 provides the benefit of minimizing harm to the human epidermis (25) as well as being a U.S. Food and Drug Administration-approved biocide used in the clinical setting regularly. In our *in vitro* MFC experiments with A. fumigatus, we found that the presence of BZK is required for NB-201 to function as a biocide. Furthermore, the presence of serum also presented an effect on the efficacy of NB-201s MFC (see Table S1 in the supplemental material). The *in vitro* susceptibility test with NB-201 was observed on every tested fungus, including C. albicans, A. fumigatus, *Mucorales*, and *Cryptococcus* spp., with an exceptional killing efficacy observed in all four serotypes of C. neoformans.

10.1128/mSphere.00729-19.5 TABLE S1 One-hour *in vitro* activity of NB-201 at various benzalkonium chloride (BZK) concentrations. Download Table S1, DOCX file, 0.04 MB. Copyright © 2019 Garcia et al. 2019 Garcia et al. This content is distributed under the terms of the Creative Commons Attribution 4.0 International license.

During our *in vitro* test, we observed a similar trend in the efficacy of NB-201. Interestingly, we observed that longer incubation times with NB-201 resulted in a lowered MFC regardless of the fungal organism that was being tested. The top etiological agent of candidiasis, C. albicans, still ranks among the leading fungal organisms to cause infection in immunocompromised patients around the world and causes >50% of bloodstream infections in the United States (3). The biofilms produced by this fungal organism make it intrinsically harder to treat and are a growing problem and concern in the clinical setting (12, 13, 26). We found that NB-201 has *in vitro* antifungal activity against planktonic organisms and biofilms of C. albicans. Furthermore, *in vitro* activity was also observed against drug-resistant clinical isolates. In an animal subcutaneous infection model, NB-201 also exhibited antifungal activity against two azole-resistant strains, TW1 and TW17 (Fig. 2, 3, and 4). These results demonstrate that NB-201 has anti-C. albicans activity both in *in vitro* and *in vivo* regardless of drug resistance. Due to the killing nature of NB-201, C. albicans is less likely to develop resistance (Fig. 2). Further tests with wound fungal infections using a porcine model or any larger animal model will provide further insights on the efficacy of NB-201.

Our *in vitro* data with other pathogenic fungi open the possibility that NB-201 can be used for other types of fungal infections. When testing NB-201 against the four serotypes of C. neoformans, we observed a dramatic killing ability. To further confirm what we observed, we employed a microscopy approach where we measured cell death with a propidium iodide stain. Within 30 min, a clear distinction of live and dead cells was observed (Fig. S2). C. neoformans is the etiological component of cryptococcosis, an infectious fungal disease known to target the respiratory tract and central nervous system in humans (27–29). Exposure to C. neoformans is common among the general population, with the majority of infectious cases resulting from reactivation due to latency in cell-mediated immunity (29). Despite advances in modern medicine, the morbidity and mortality for C. neoformans infections remain unacceptably high, with mortality rates of up to 20% in infected AIDS patients (27). C. neoformans is resistant to the newest antifungal drug class, echinocandins. The infection primarily afflicts the lungs; therefore, respiratory treatments of nanoemulsions could be applied. Although NB-201 may not be suitable for this due to potential toxicity of the surfactant BZK to the lungs, a lung-safe NE could be explored for a novel form of treatment for respiratory cryptococcosis infection.

Known as one of the most prevalent airborne fungal pathogens in the world, A. fumigatus causes invasive aspergillosis (IA) in immunocompromised patients (30). In immunocompetent individuals, IA is able to be naturally combated by the natural immunosuppressive abilities of the human body (30). A. fumigatus primarily infects the respiratory tract (31). Thus, as in the case of C. neoformans, further developing a lung-safe NE would be of interest. Interestingly, amphotericin B releasing topical nanoemulsions for the treatment of aspergillosis and candidiasis has been developed and showcases the versatile potential of utilizing nanoemulsions (32).

Mucormycosis is a recently emerging opportunistic fungal infection (33). The typical causative agents for mucormycosis fall under the *Mucorales* family, which include *Rhizopus* spp., *Mucor* spp., and others (34). Mucormycosis presents itself with a mortality rate of ∼50% in all mucormycosis cases (35–37). Following the theme we have observed previously, NB-201 presented a killing ability comparable to that observed in our C. albicans *in vitro* experiments. Mucormycosis is typically caused when the acquired spores are inhaled into the body (35, 36). Cutaneous infections of mucormycosis in patients undergoing traumatic injury have been reported (35, 38), and in the event that patients survive infection, they typically suffer from disfiguration due to surgical debridement of infected tissue, a common way of treating mucormycosis infection aside from treatment with amphotericin B (39). NB-201 therefore presents itself as an option in combating *Mucorales* infection.

Due to the nature of NB-201, the use of it as a topical treatment alternative for fungal infections, with little to no drug resistance being developed by the fungi it is killing, could be a possibility. BZK in NB-201 can pose detrimental effects to the lungs, and intravenous administration cannot be used due to lysis of NE. Thus, it exhibits limited application, such as topical or subcutaneous treatment. Such treatments could include ointments for skin infections and even oral washes for possible oral-pharyngeal infections. Other possible uses for NB-201 are to treat dermatophyte infections and oral or vaginal candidiasis, but these uses warrant further investigations.

### Conclusions.

Cases of immunocompromised patients being infected with antifungal drug-resistant fungi have been rising. The development of drug resistance and the limited availability of antifungal drugs have left us in a scenario where we need to develop new therapeutic approaches that are less prone to the development of resistance by pathogenic fungi. Previously, NB-201 presented a highly effective antimicrobial potential against various methicillin-resistant S. aureus strains both *in vitro* and *in vivo*. In this study, we have presented a novel use for the NB-201 nanoemulsion formulation that presents killing abilities observed *in vitro* against 35 different fungi, 30 of which are either clinical isolates or antifungal drug-resistant strains. We also observed reduction in inflammation, wound healing, and fungal pathogen clearing abilities of NB-201 in a murine host model (Fig. 4). Due to the nature of the activity NB-201 presents, there is a minimized chance of drug resistance developing, presenting a novel way to control fungal wound or skin infections.

## MATERIALS AND METHODS

### Fungal strains and growth conditions.

The strains used in this study are listed in Table 1. C. albicans and C. neoformans strains were grown in liquid or solid yeast extract peptone dextrose (YPD; 10 g/liter yeast extract, 20 g peptone, 20 g dextrose, 20 g agar [for plates only]) at 30°C. *Mucorales* strains were grown in potato dextrose agar (PDA; 4 g/liter potato starch, 20 g/liter dextrose, 15 g/liter agar) or yeast extract peptone glucose agar (YPG; 3 g/liter yeast extract, 10 g/liter peptone, 20 g/liter glucose, 2% agar, pH 4.5) at 30°C in the light for 4 days. A. fumigatus strains were grown in PDA at 30°C for 4 days. To collect spores of *Mucorales* and A. fumigatus, sterile water (2 ml per plate) was added to the plate and spores were collected by gently scraping the fungal mycelial mats.

### *In vitro* efficacy of NB-201 against C. albicans planktonic cells and biofilms.

The initial concentration of NB-201 was 10% for all experiments in this study, except for the data shown in Table S1 in the supplemental material, where a different concentration of NE was tested against A. fumigatus. The C. albicans strains were inoculated at a concentration of 1 × 10^6^ in a 96-well plate containing NB-201 serially diluted in RPMI (100 μl per well), ranging from 1:1 to 1:2,048. Ten-microliter samples were taken at 1, 24, 48, and 72 h from each well and plated on PDA agar plates, which were incubated for 48 h. After incubation, every plate was examined for growth on the site of inoculation. The lowest concentration (diluted ratio) at which no colonies form on PDA was defined as the minimum fungicidal concentration (MFC). For biofilms, C. albicans was plated at a concentration of 1 × 10^6^ in a 96-well plate containing RPMI medium and incubated for 24 h to form a biofilm on the bottom of the wells. After biofilm formation, the RPMI medium was removed and the biofilms were washed with PBS. The medium then was replaced with medium containing NB-201 serially diluted in PBS concentrations ranging from 1:1 to 1:2,048. The medium containing the nanoemulsion treatment then was removed at 2-, 4-, 6-, 24-, 48-, and 72-h time points. The biofilms were washed with PBS and stained with 0.6% crystal violet stain. The biofilms then were washed one more time with PBS to remove any residual crystal violet stain. Finally, the biofilms were destained with 33% acetic acid, and 85 μl of the supernatant was transferred to a clean 96-well plate. The destained crystal violet supernatant then was read on a plate reader and quantified.

In a fashion similar to that of the crystal violet assay, C. albicans was plated in a 96-well plate containing RPMI medium at a concentration of 1 × 10^6^ and incubated for 24 h to form a biofilm on the bottom of the wells. After a biofilm was formed, the RPMI medium was removed and the biofilms were washed with PBS. The medium then was replaced with medium containing NB-201 serially diluted at concentrations ranging from 1:1 to 1:2,048, as diluted in PBS. The medium containing the nanoemulsion treatment then was removed at 2-, 4-, 6-, 24-, 48-, and 72-h time points, and the biofilms were washed with PBS to remove any nonadherent cells. The biofilms then were treated with 100 μl of XTT solution containing 3.5 μl of menadione and incubated for 2 h at 37°C. Eighty-five microliters of the supernatant was transferred to a clean 96-well plate, read in a plate reader, and then quantified. All *in vitro* efficacy experiments were repeated at least twice to verify the results.

### *In vitro* efficacy of NB-201 against *Mucorales* spp., C. neoformans, and A. fumigatus.

The respective fungal strains were inoculated at a concentration of 1 × 10^6^ in a 96-well plate containing NB-201 serially diluted in RPMI (100 μl per well). Dilution concentrations ranged from 1:1 to 1:2,048. Ten-microliter samples were taken at 1, 24, 48, and 72 h from each well and plated on PDA agar plates, which were incubated for 48 h. After incubation, every plate was examined for growth on the site of inoculation.

### *In vivo* efficacy of NB-201 in a murine subcutaneous infection model.

CD-4 mice weighing between 19 and 23 g were housed together. C. albicans strains SC5314, TW1, and TW17 were grown in YPD liquid medium, washed in PBS, and suspended in PBS at a concentration of 1 × 10^6^. Under anesthesia, the dorsal fur of the mice was shaved. The exposed skin was washed with 70% ethanol, and mice were infected with 1 × 10^6^ CFU via subcutaneous injection on the shaved dorsal side. Subsequent subcutaneous injections of NB-201, PBS, or fluconazole followed at 6, 24, and 48 h. The mice were euthanized at 72 h, and the skin of the infected area was collected immediately for analysis.

The collected mouse tissues were weighed and placed in PBS on ice. The tissues then were homogenized with a tissue homogenizer. The homogenized tissue then was diluted 1:10 and plated on YPD agar plates treated with antibiotics to prevent unwanted bacterial growth. The plates were incubated at 37°C for 48 h (19, 20). The numbers of CFU per gram of tissue were determined. A Student's *t* test was carried out for all statistical analysis to evaluate the *in vivo* efficacy of NB-201 on subcutaneous infection. A *P* value of ≤0.05 was considered significant for this study.

Animals were sacrificed and their skin tissue excised. The tissue samples were immersed in a formalin fixative agent. The tissue blocks were processed for cryosectioning. Ten- to 12-μm-micron thick sections were obtained with a cryostat and stained with hematoxylin and eosin for histopathological examination. Observations were made under a light microscope, and representative photomicrographs at ×10 and ×20 magnification were used for comparative study.

All murine experiments were conducted at the University of Texas at San Antonio (UTSA) in full compliance with all of the guidelines of the UTSA University Institutional Animal Care and Use Committee (IACUC) and in full compliance with the United States Animal Welfare Act (Public Law 98–198). The UTSA IACUC approved all of the murine studies under protocol number MU104-02-20. The experiments were conducted in the Division of Laboratory Animal Resources (DLAR) facilities, which are accredited by the Association for Assessment and Accreditation of Laboratory Animal Care (AAALAC).

### Toxicity evaluation of NB-201 *in vitro* using mammalian cell types.

Cell cytotoxicity determinations were performed using an automated luminescence assay based on the luciferin reaction. Recombinant luciferase (Promega Corp., Madison, WI) was used to catalyze the conversion of luciferin substrate to oxyluciferase and light in the presence of ATP and other cofactors, including Mg^2+^ and molecular oxygen. Thus, the assay detects ATP produced by metabolically active viable cells, yielding a luminescent signal that is directly proportional to the total number of cells per well in a 384-well format. The assay was used to screen new nanoemulsion compounds for cell toxicity on murine macrophage (Raw264.7), epithelial cell (TC-1), and dendritic cell (Jaws II) lines. The 50% inhibitory concentration (IC_50_) for each formulation was calculated after 24 h of NE exposure. The IC_50_ is defined as the nanoemulsion concentration (percent, wt/wt) yielding 50% inhibition at 24 h for each cell line. For the formulation of NB-201, the IC_50_ was 0.073% in TC-1 cells.
