# Supplementary figures and images for: Nanoemulsion as an Effective Treatment against Human-Pathogenic Fungi
Source: mSphere. 2019 Dec 18;4(6):e00729-19. doi: 10.1128/mSphere.00729-19 (PMC6920514; doi:10.1128/mSphere.00729-19)

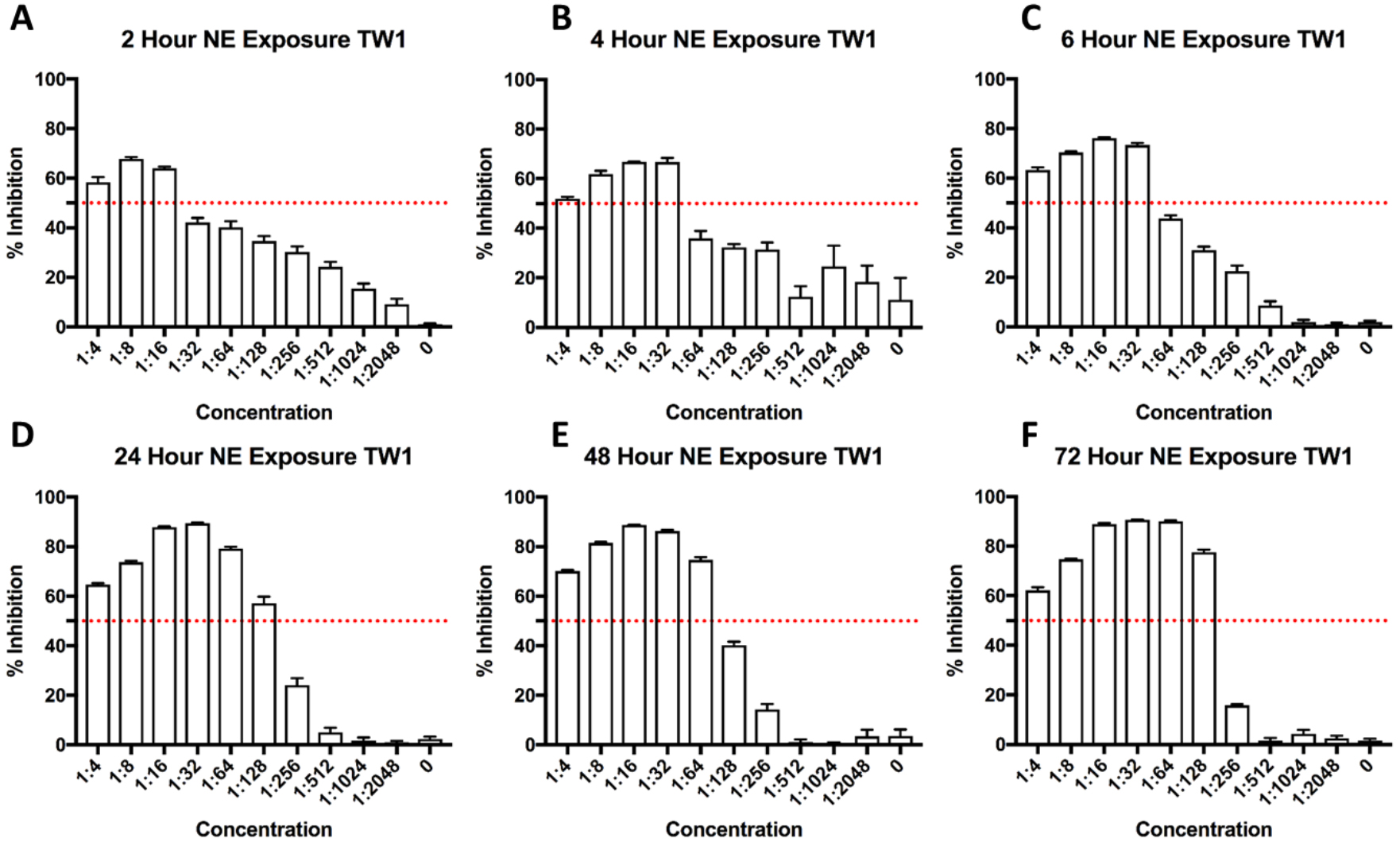

Supplement: FIG S1 [file mSphere.00729-19-sf001.jpg]

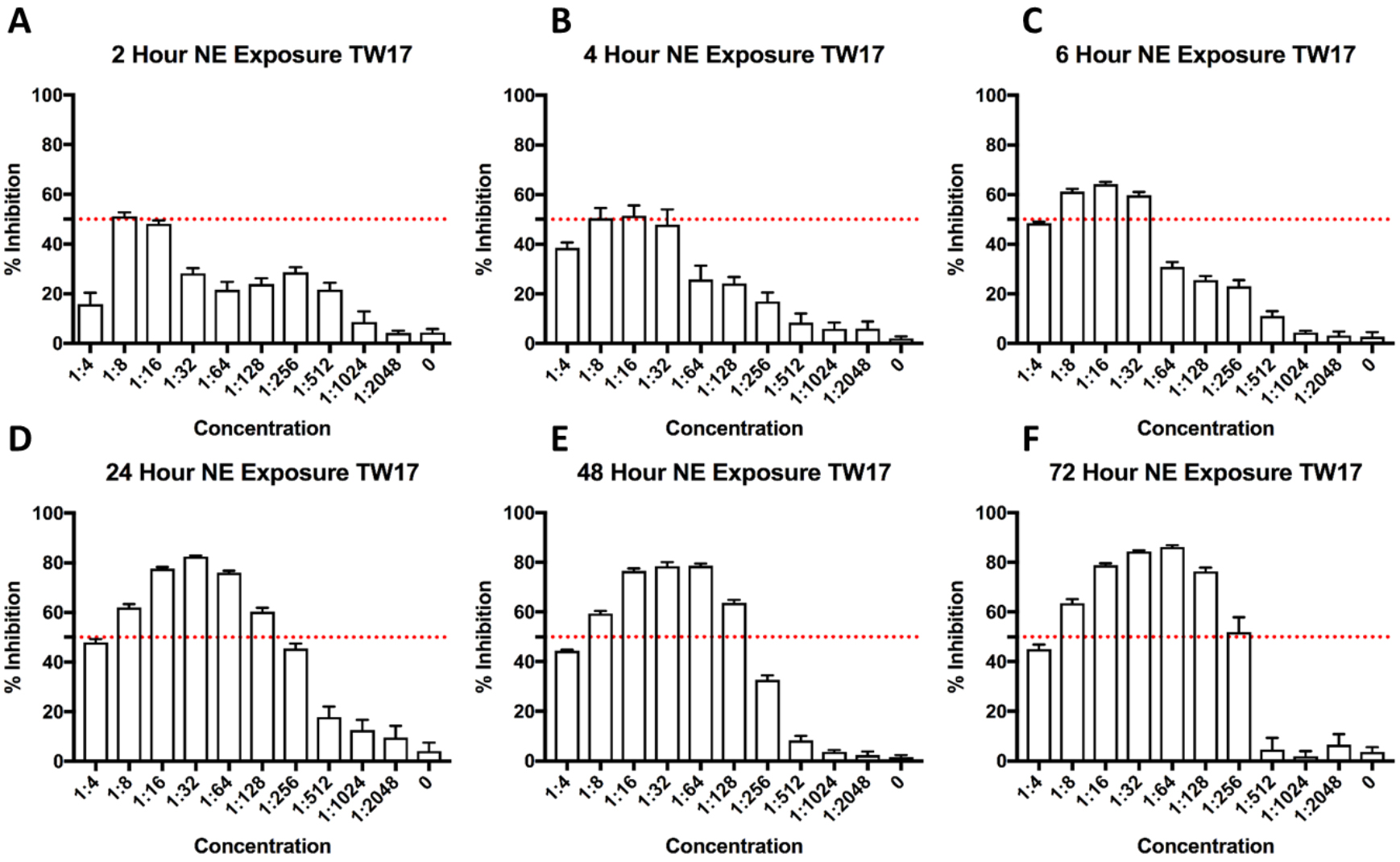

Supplement: FIG S2 [file mSphere.00729-19-sf002.jpg]

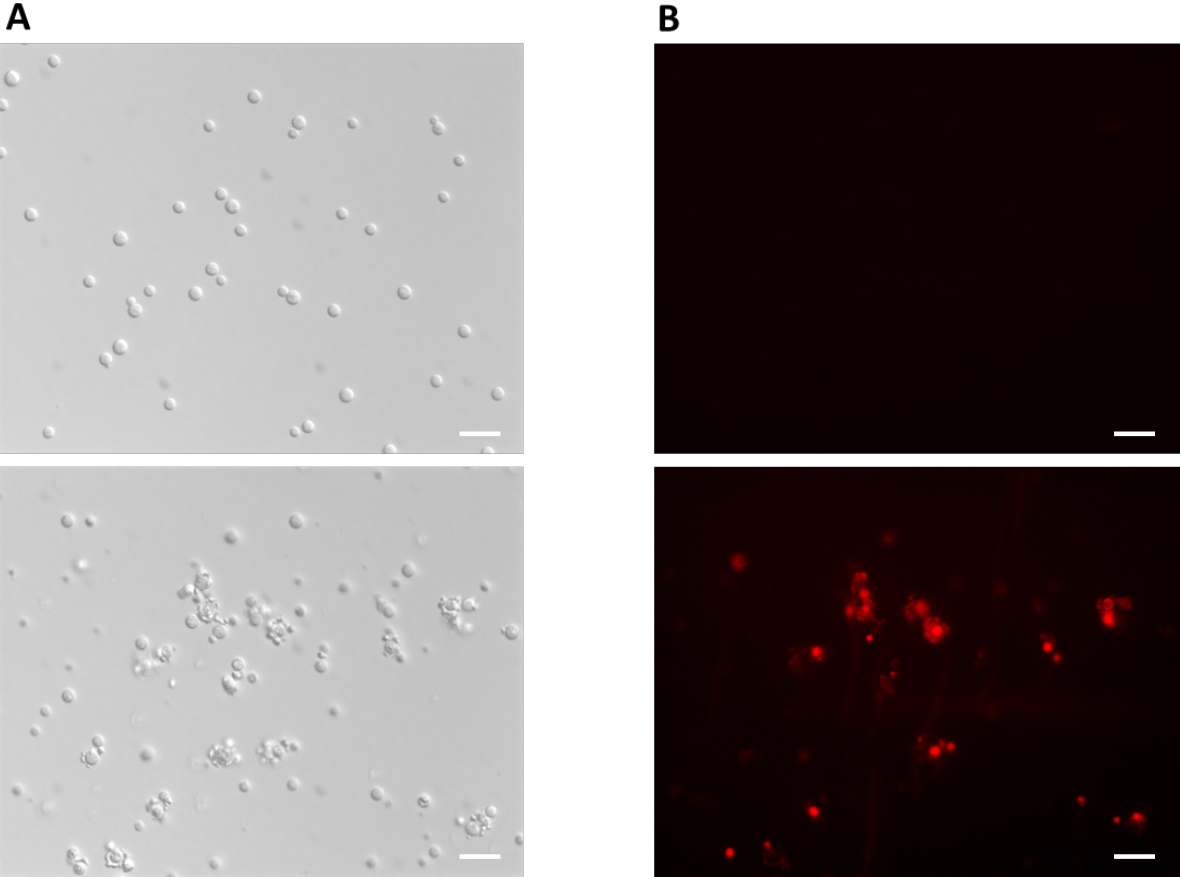

Supplement: FIG S3 [file mSphere.00729-19-sf003.jpg]

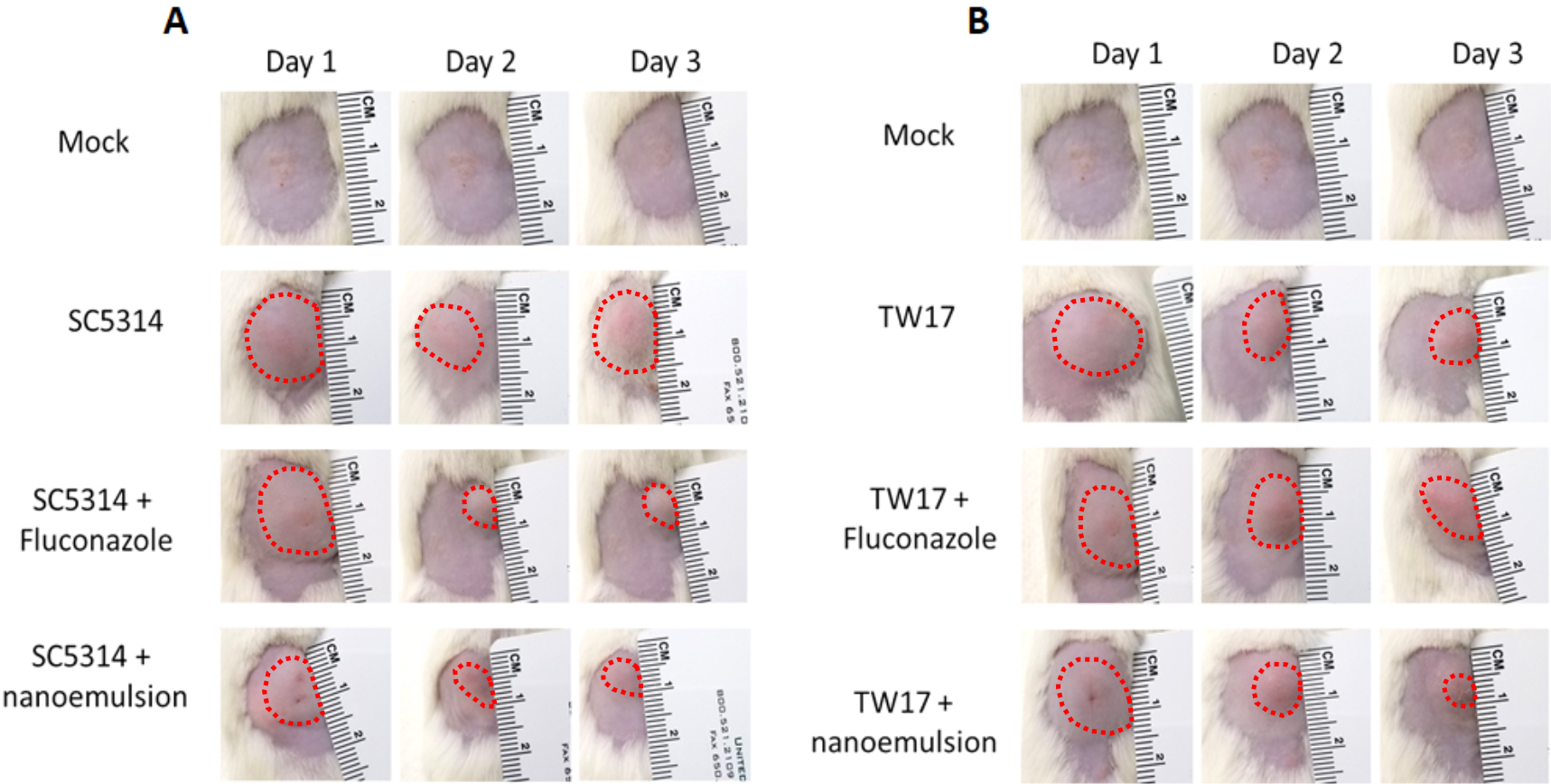

Supplement: FIG S4 [file mSphere.00729-19-sf004.jpg]
