# Supplementary material for: Nanoemulsion as an Effective Treatment against Human-Pathogenic Fungi
Source: mSphere. 2019 Dec 18;4(6):e00729-19. doi: 10.1128/mSphere.00729-19 (PMC6920514; doi:10.1128/mSphere.00729-19)
Supplement: TABLE S1 [file mSphere.00729-19-st001.docx]

| Nanoemulsion | | | MIC | |
| --- | --- | --- | --- | --- |
| **NE%** | **BZK%** | **EDTA%** | **No Serum** | **25% Serum** |
| 40 | 0.8 | 0.74 | 1:64 | 1:16 |
| 20 | 0.4 | 0.74 | 1:32 | 1:16 |
| 10 | 0.2 | 0.74 | 1:16 | 1:8 |
| 10 | 0 | 0 | No inhibition | No inhibition |
